# Factors Associated With Smoking Status among HIV-Positive Patients in Routine Clinical Care

**DOI:** 10.4172/2155-6113.1000480

**Published:** 2015-07-09

**Authors:** Cosmas M Zyambo, James H Willig, Karen L Cropsey, April P Carson, Craig Wilson, Ashutosh R Tamhane, Andrew O Westfall, Greer A Burkholder

**Affiliations:** 1 Department of Epidemiology, School of Public Health, University of Alabama at Birmingham, USA; 2 Division of Infectious Diseases, School of Medicine, University of Alabama at Birmingham, USA; 3 Department of Public Health, School of Medicine, University of Zambia, Zambia; 4 Department of Biostatistics, School of Public Health, University of Alabama at Birmingham, USA; 5 Department of Psychiatry, School of Medicine, University of Alabama at Birmingham, USA

**Keywords:** Smoking, HIV, Risk factors

## Abstract

**Background:**

Treatment-related reductions in morbidity and mortality among human immunodeficiency virus (HIV)-positive patients have been attenuated by cigarette smoking, which increases risk of cardiovascular, respiratory, and neoplastic diseases. This study investigated factors associated with smoking status among HIV-positive patients.

**Methods:**

This cross-sectional study included 2,464 HIV-positive patients attending the HIV Clinic at the University of Alabama at Birmingham between April 2008 and December 2013. Smoking status (current, former, never), psychosocial factors, and clinical characteristics were assessed. Multinomial logistic regression was used to obtain unadjusted and adjusted odds ratios (OR) and 95% confidence intervals (CI) for the association of the various factors with smoking status.

**Results:**

Among HIV-positive patients (mean age 45 years, 75% male, 55% African-American), the majority reported a history of smoking (39% current and 22% former smokers). In adjusted models, patient characteristics associated with increased odds of current smoking were male gender (OR for heterosexual men, 1.8 [95% CI: 1.3–2.6]; for men who have sex with men, 1.5 [1.1–1.9]), history of respiratory diseases (1.5 [1.2–1.9]), unsuppressed HIV viral load (>50 copies/mL) (1.5 [1.1–1.9]), depression (1.6 [1.3–2.0]), anxiety (1.6 [1.2–2.1]), and prior and current substance abuse (4.7 [3.6–6.1] and 8.3 [5.3–13.3] respectively). Male gender, anxiety, and substance abuse were also associated with being a former smoker.

**Conclusions:**

Smoking was common among HIV-positive patients, with several psychosocial factors associated with current and former smoking. This suggests smoking cessation programs in HIV clinic settings may achieve greater impact by integrating interventions that also address illicit substance abuse and mental health.

## Background

While the life expectancy of persons with human immunodeficiency virus (HIV) has increased with effective antiretroviral therapy (ART) [1–3], smoking remains an important health risk. Currently, smoking is the leading preventable cause of morbidity and mortality in the United States (US) [4], and it is a risk factor for many chronic diseases including cardiovascular disease (CVD), chronic obstructive pulmonary disease (COPD), and numerous types of cancer [5–7]. In the US, tobacco-related diseases account for approximately 480,000 deaths and are directly responsible for national healthcare expenditures and productivity loses of approximately $289 billion every year [8]. While an estimated 17.8% (42.1 million) of US adults smoke [9], the prevalence of smoking among HIV-positive individuals is 2–3 times higher [10–13].

The higher prevalence of smoking among HIV-positive patients has significant public health implications including the higher mortality rates among smokers [14–16]. In a study of US veterans, Crothers et al. showed that the mortality rates per 100 person-years were 1.76 for HIV-negative patients who had never smoked, 2.45 for HIV-positive patients who had never smoked, and 5.48 for HIV-positive patients who were current smokers [15]. In addition, smoking is a contributor to the higher rates of cardiovascular events, COPD, and lung cancer observed in HIV-positive patients compared to uninfected persons [17–19]. These findings raise the concern that although advances in HIV treatment have resulted in morbidity and mortality reductions, the gains of ART may be attenuated by high rates of cigarette smoking.

Research on factors associated with current smoking among HIV-positive patients may assist health care providers to implement targeted smoking cessation programs addressing smoking and these other factors. Most previous studies investigating factors associated with smoking among HIV-positive patients have used small sample sizes or were clinical trials [12,20–22]. Therefore, the purpose of the current study was to investigate socio-demographics, psychosocial factors, and clinical characteristics associated with smoking status among a large population of HIV-positive patients in a routine clinical setting in the southeastern US.

## Methods

### Study setting

This cross-sectional study was conducted using data from the University of Alabama at Birmingham (UAB) 1917 HIV Clinic Cohort protocol, which has prospectively collected detailed socio-demographic, psychosocial, and clinical data on HIV-positive patients receiving primary HIV and subspecialty care at the UAB 1917 HIV/AIDS Clinic (1917 Clinic) since 1992 (N >8,000 patients overall; >3,000 active). This study was approved by the UAB Institutional Review Board.

### Eligibility criteria

All HIV-positive patients, aged ≥19 years, who attended the outpatient clinic between April 2008 and December 2013 and who completed a self-administered survey during the index visit were eligible. This survey was not available prior to April 2008. The index visit was the most recent patient visit between April 2008 and December 2013. A total of 2,528 HIV-positive patients met the eligibility criteria. However, 2.5% (63) were excluded from the analysis because they did not answer any of the tobacco use questions in the survey. Patients of race/ethnicity other than Caucasian or African-American were excluded for low numbers. 2,464 HIV-positive patients were included in the final analysis.

### Data sources

Socio-demographics, diagnoses, medications, and laboratory data were obtained by electronic data query of the 1917 Clinic Cohort database using MS SQL Server 2008. The 1917 Clinic uses a patient-reported outcomes (PRO) software capture system to collect psychosocial information from patients. The system employs standardized and validated questionnaires that are self-administered via computer touchscreens during patient visits. The questionnaires include assessments on smoking, depression (Patient Health Questionnaire-9 [PHQ-9]), anxiety (Patient Health Questionnaire-Anxiety [PHQ-Anxiety]), substance use (Alcohol, Smoking, and Substance Involvement Screening Test [ASSIST]), and ART adherence (Adult AIDS Clinical Trials Group [AACTG] Adherence Instrument) [23].

### Variables

#### Outcome variable

The outcome of interest was smoking status, which was categorized as never smoker, former smoker, or current smoker as defined by the tobacco use questionnaire. Patients were classified as “current smokers” if they answered “yes” to the question, “*Do you currently smoke cigarettes*?” Patients who reported having smoked more than 20 cigarettes in their lifetime but who were not current smokers were classified as “former smokers”. Those who did not meet the criteria for current or former smokers were classified as “never smokers”.

#### Independent variables

The independent variables collected were: 1) Socio-demographics including age, composite gender/sexual orientation (men who have sex with men [MSM], heterosexual men, and women), and race (Caucasian, African-American). 2) Psychosocial factors including depression (major or other depressive symptoms based on PHQ-9 scoring); anxiety (symptoms of anxiety or panic syndrome based on PHQ-Anxiety scoring), and substance abuse as per the ASSIST (opioids, marijuana, crack/cocaine, amphetamines). 3) Clinical variables including ART adherence (missing ≥ 1 dose over the previous 1 month using the AACTG Adherence instrument was considered non-adherent), history of cardiovascular diseases (stroke, myocardial infarction, coronary heart disease [CHD], and hypertension), history of respiratory diseases (asthma, COPD, and bacterial pneumonia), history of metabolic diseases (diabetes and dyslipidemia), and laboratory values (closest CD4 count and plasma HIV-1 RNA [viral load; VL]). A history of comorbidity was defined as ever being diagnosed with that comorbidity on or prior to the index visit. Laboratory values from the index visit or prior to the index visit were used.

### Statistical analyses

The variables in the study were evaluated using descriptive statistics including chi-square and analysis of variance (ANOVA) as appropriate. Continuous variables are reported as means with standard deviations (SD) and categorical variables as frequencies with percentages. Multinomial (polytomous) logistic regression was used to examine the factors associated with current and former smoking in unadjusted and adjusted models, using never smokers as the referent. Odds ratios (OR) and 95% confidence intervals (CI) were obtained from univariate and multivariable models. All the variables were felt to be clinically relevant and included in the adjusted model. Statistical significance was set at the 0.05 level (two-tailed test). Analyses were performed using SAS version 9.3 (SAS Institute, Cary, NC).

## Results

Overall, 39% of HIV-positive patients were current smokers and 22% were former smokers (Table 1). The mean age (± standard deviation) was 45 ± 11 years and the population was predominantly African American (55%). The majority of the patients were male (75%), and 59% were MSM. HIV VL was detectable (≥ 50 copies/mL) in 29% of patients, and 12% of patients had a CD4 count of <200 cells/μL. Cardiovascular diseases (48%), metabolic diseases (41%), and respiratory diseases (23%) were common overall. Among patients with history of respiratory diseases, current smoking was more prevalent than among those with history of cardiovascular and metabolic diseases. For psychosocial factors, 49% of patients with depression, 51% of patients with anxiety, and 73% of current substance users were also current smokers.

### Factors associated with current and former smoking

In the univariate analyses, male heterosexual, MSM, ART non-adherence, history of respiratory disease, detectable HIV VL (>50 copies/mL), depression, anxiety, and current and prior substance abuse were significantly associated with increased odds of being a current smoker (Table 2). In contrast, African-American race, and history of cardiovascular or metabolic disease were associated with significantly decreased odds of current smoking. Older age, male heterosexual, MSM, history of metabolic disease or cancer, depression, anxiety, and current and prior substance abuse were significantly associated with increased odds of being a former smoker. African-American race was associated with significantly decreased odds of being a former smoker.

In adjusted analyses, factors significantly associated with being a current smoker included male heterosexual (OR, 1.8 [95% CI: 1.3–2.6]), MSM (1.5 [1.1–1.9]), history of respiratory disease (1.5 [1.2–1.9]), detectable VL (1.5 [1.1–1.9]), depression (1.6 [1.3–2.0]), anxiety (1.6 [1.2–2.1]), prior substance abuse (4.7 [3.6–6.1]), and current substance abuse (8.3 [5.3–13.3]) (Table 2). African-American race (0.7 [0.6–0.9]) and history of cardiovascular disease (OR, 0.8 [0.6–0.9]) or metabolic diseases (OR, 0.8 [0.6–0.9]), were associated with significantly decreased odds of being a current smoker. Factors which were significantly associated with former smoking included older age (OR per 10 year increase, 1.3 [1.2–1.5]), male heterosexual (2.3 [1.5–3.2]), MSM (1.7 [1.2–2.4]), anxiety (1.5 [1.1–2.0]), prior substance abuse (3.2 [2.5–4.3]), and current substance abuse (2.6 [1.4–4.6]), whereas African-American race was associated with significantly decreased odds of former smoking (0.6 [0.5–0.8]).

## Discussion

In this population of HIV-positive patients receiving routine clinical care, 39% of patients reported being current smokers. This estimate is slightly lower than what has been reported in previous studies among HIV-positive persons (40–59%) [10–13], yet it remains notably higher than the smoking prevalence found in the general US population (20.6%) [10] and in Alabama (22%) [24]. Twenty-two percent of the HIV-positive patients in our study population were former smokers, and compared with current smokers, they were less likely to have depression or be current substance abusers. This suggests that there may be opportunities for combining smoking cessation programs with targeted interventions to address mental health and substance abuse issues among current smokers with HIV who have these comorbidities.

In the context of numerous studies that have demonstrated an association of mental illness with cigarette smoking among both the general population and HIV-infected patients [10, 25–27], it is not surprising that roughly half of our patients with depression and anxiety were current smokers. A potential reason for the high co-occurrence of current smoking with mental illness is that some patients may smoke to cope with psychological distress, including the stress of living with HIV [25,27].

Our study examined overall substance abuse, including crack/cocaine, marijuana, amphetamine and opioids, and found that substance abuse was strongly associated with current and former smoking status, similar to findings reported in prior studies among both the general population and HIV-infected patients [10,28,29]. The association between current substance abuse and current smoking was especially strong. Similar social, genetic, and personality factors are associated with smoking and substance abuse [30–33]. In addition, there are similarities in the neurophysiology of addiction to nicotine and illicit drugs [34]. Smoking cessation interventions among both the general population and HIV-positive patients have traditionally addressed smoking independent of other comorbidities. However, given the correlations of mental illness and substance abuse with current smoking among HIV-positive patients observed in our study and others, an important approach toward smoking cessation in routine HIV care settings might be to address smoking cessation as part of the care offered for these comorbid conditions, as has been advocated by some for the general population [35]. Such approaches may be of particular importance among HIV-positive patients given that there is higher prevalence of mental illness and substance abuse in this population which likely contribute significantly to higher rates of smoking [10,27]. Of note, recent studies suggest readiness to quit among smokers with depression and substance abuse does not differ significantly from smokers without these conditions and thus it is reasonable to pursue smoking cessation interventions among these patients [22,35].

After adjusting for socio-demographic, clinical, and psychosocial factors, our analyses showed that patients with detectable HIV VL (>50 copies/mL) were significantly more likely to be current smokers. This finding is similar to results from other studies that demonstrated that current smoking status was associated with failed virological suppression for patients on ART [36,37]. However, this association was not present among former smokers in our study. A potential explanation may be that current smokers are less likely to engage in self-care behaviors such as ART adherence compared with former or never smokers. In our study, patients who were non-adherent to ART were more likely to be current than former or never smokers, however, in our multinomial logistic regression analyses, no significant association was found between ART adherence and smoking status. The proportion of patients non-adherent to ART in our study was low (12%), limiting our ability to make broad inferences about this segment of our population.

A history of respiratory diseases (asthma, COPD, and bacterial pneumonia) was significantly associated with increased odds of current smoking in our study. Studies have shown that respiratory diseases, such as COPD, are common among HIV-positive patients, occurring at higher rates than observed in uninfected persons, and may have a more accelerated course [18,38,39]. While smoking is a strong contributor to COPD among HIV-infected patients, an excess risk remains compared to uninfected persons even when adjusting for smoking [39,40]. Persons with HIV appear to be more susceptible to the damaging effects of smoking [18,41]. The pathophysiology is incompletely understood, however hypotheses have been advanced suggesting that tissue inflammatory processes, altered antioxidant-oxidant balance, and increased apoptosis of the lung epithelial and endothelial cells play a role [39]. Smokers with concomitant respiratory co-morbidities should be aggressively targeted in smoking cessation interventions in HIV care settings.

Smoking is a risk factor for cardiovascular diseases in both general and HIV-positive populations [11,14]. In our cohort, patients with a history of cardiovascular diseases (stroke, myocardial infarction, CHD, or hypertension) were significantly less likely to be current smokers. A similar lower odds of current smoking was observed in patients with metabolic diseases. One possible explanation is that patients diagnosed with these disorders may be more motivated to quit smoking or receive more smoking cessation counseling from HIV providers or other physicians [42,43]. If this is correct, it suggests increased focus on subpopulations at risk can lead to lower smoking rates in the HIV care setting.

Our study participants were predominantly African-American and male. Studies have demonstrated that African-Americans have about the same prevalence of smoking as Caucasians but seemingly more difficulty with quitting smoking. This may be due to socioeconomic disadvantages such as being less likely to receive smoking cessation counseling and treatment [44,45]. However, in our population, African Americans were less likely to be current or former smokers, even when adjusting for gender. This finding was unexpected and bears further study. Our participants were predominantly MSM. Prior studies have shown higher smoking rates among MSM than heterosexual men in the general population as well as among HIV-positive patients [9,46,47]. In contrast, in our study prevalence of current smoking was similar between MSM and heterosexual males.

One of the major strengths of this study is the large sample size. Our study involved HIV-positive patients from a routine clinical care setting. These patients tend to differ from those sampled in clinical trials and often represent a more diverse population due to the absence of enforcement of any enrollment criteria. Most prior studies evaluating patient characteristics related to smoking among HIV-positive patients have had small sample sizes or were clinical trials. An exception is a recent study by Mdodo et al. including a large population of HIV-positive patients participating in the nationally distributed Medical Monitoring Program [10]. Our findings on the association of depression and substance abuse with smoking among HIV-positive patients corroborate the findings of this study. We used patient-reported data in our analyses, which have been shown to be more accurate in many sensitive domains [48,49], but may be biased because of self-reporting. Although self-reporting often underestimates the prevalence of smoking [50], other studies have shown that computer-administered questionnaires have comparable validity with clinical interviews [51]. Finally, our analyses included medical comorbidities, which have been absent from most prior studies.

Our results should also be interpreted with regard to the limitations of our study. Because of the observational design, we only were able to identify the factors associated with smoking status and were unable to address causality. We were unable to assess changes in these factors over time. Despite adjusting for known confounders in the multivariable model, the potential for the residual confounders inherent in observational studies remains and might affect the interpretation of study outcomes. We analyzed data of patients who completed the PROs during the study period and lack information on the patients who did not take part. However, we have low concern that this has biased our results as >90% of our patients consent to the PROs. Although the tobacco use questionnaire contains questions regarding number of cigarettes smoked per day and years a patient has smoked, there was >60% missing data for these questions; thus we could not evaluate whether there was a dose-effect relationship between patient characteristics and pack-years of smoking. Some of our effect sizes are modest and thus may potentially be statistically significant without representing clinical significance. However we note that our findings on the associations of male gender, respiratory diseases, detectable HIV viral load, depression, anxiety, and substance abuse with smoking are consistent with prior studies among HIV-positive patients. Family history and heritability have been shown to play an important role in smoking, substance abuse, and mental illness, however we lacked data on family history [52–54]. However, we note our ability to control for a number of important sociodemographic, clinical, and behavioral factors available through our cohort database and PRO platform. Finally, one cross-sectional study from a single site has limited generalizability to other national or international settings.

## Conclusion

Based on our findings, smoking cessation programs in HIV clinic settings may achieve greater impact by integrating interventions to address illicit substance use and mental illness into their protocols given the high co-occurrence of these conditions. Given the impact of smoking on morbidity and mortality among HIV-positive patients, and its attenuating effects on the benefits afforded by ART, the development and implementation of effective smoking cessation programs is a high priority in this population.

## Figures and Tables

**Table 1:** Characteristics of HIV-positive patients receiving routine care at the UAB 1917 HIV/AIDS clinic from April 2008–December 2013 by smoking status.

	Overall	Never smokers	Former smokers	Current smokers
Variables	(N = 2,464)	(n = 970)	(n = 538)	(n = 956)
	n (%)^a^	n (%)^b^	n (%)^b^	n (%)^b^
**Sociodemographic**				
Age (years), mean (SD)	45.4 (11.4)	44.5 (11.8)	48.2 (11.3)	44.6 (10.7)
Gender/sexual orientation				
Women	542 (22.0)	267 (49.3)	86 (15.9)	189 (34.8)
Male heterosexual	405 (16.4)	146 (36.0)	101 (24.9)	158 (39.0)
MSM	1447 (58.7)	542 (37.5)	332 (22.9)	573 (39.5)
Race/ethnicity				
Caucasian	1,090 (45.4)	328 (30.0)	295 (27.1)	467 (42.9)
African American	1,310 (54.6)	612 (46.7)	228 (17.4)	470 (35.9)
**Clinical**				
ART Adherence				
Non-adherent	295 (12.0)	103 (34.9)	49 (16.6)	143 (48.5)
*Comorbidities* c				
Cardiovascular	1,182 (48.0)	499 (42.2)	292 (24.7)	391 (33.1)
Respiratory	555 (22.5)	198 (35.7)	114 (20.5)	243 (43.8)
Metabolic	1,009 (40.9)	413 (40.9)	267 (26.5)	329 (32.6)
Any cancers	280 (11.4)	107 (38.2)	80 (28.6)	93 (33.2)
*Laboratory parameters* d				
Viral Load (copies/mL)				
Detectable (≥ 50)	702 (28.6)	242 (35.5)	131 (18.7)	329 (46.9)
CD4 count (cells/μL)				
<200	297 (12.1)	120 (40.4)	54 (18.2)	123 (41.4)
200–350	367 (15.0)	145 (39.5)	86 (23.4)	136 (37.1)
>350	1,789 (72.9)	701 (39.2)	396 (22.1)	692 (38.7)
**Psychosocial**				
Depression	992 (40.8)	299 (30.1)	207 (20.8)	486 (48.9)
Anxiety	580 (24.0)	154 (26.6)	128 (22.1)	298 (51.4)
Substance abuse e				
Never	1,512 (61.1)	804 (53.2)	294 (19.4)	414 (27.3)
Prior	733 (29.9)	134 (18.3)	212 (28.9)	387 (52.7)
Current	210 (8.6)	27 (12.9)	30 (14.3)	153 (72.9)

Abbreviations: ART- Antiretroviral Therapy; HIV- Human Immunodeficiency Virus; MSM- Men who have sex with men; SD- Standard Deviation; UAB- University of Alabama, Birmingham

Comorbidities: Cardiovascular (stroke, myocardial infarction, coronary artery disease, hypertension); Respiratory (asthma, chronic obstructive pulmonary disease, bacterial pneumonia); Metabolic (diabetes and dyslipidemia);

Missing data: Gender/sexual orientation, 2.8 %; adherence, 0.6%; Race, 2.6%; viral load, 0.4%; depression (PHQ-9), 1.3%; anxiety (PHQ-A), 1.7%; substance abuse (ASSIST), 1.7%

All the variables were significant at p value <0.05 level except for CD4 count

a Column percentages;

b row percentages;

c Yes to history of comorbidities;

d Lab value closest on or prior to index visit;

e Substance abuse includes (opioids, marijuana, crack/cocaine, amphetamines)

**Table 2:** Multinomial logistic regression model examining the factors associated with current and former smoking among HIV-positive patients receiving care at the UAB 1917 HIV/AIDS clinic April 2008–December 2013 (N = 2,464).

	Smoking status			
	Former smokers		Current Smokers	
	Unadjusted	Adjusted	Unadjusted	Adjusted
	OR (95% CIs)	OR (95% CIs)	OR (95% CIs)	OR (95% CIs)
**Sociodemographics**				
Age (per 10 years) a	1.3 (1.2–1.5)	1.3 (1.2–1.5)**	1.0 (0.9–1.1)	1.0 (0.9–1.2)
Gender/sexual orientation				
Women	1.0	1.0	1.0	1.0
Male heterosexual	2.1 (1.5–3.0)	2.3 (1.5–3.2)**	1.5 (1.1–2.0)	1.8 (1.3–2.6)**
MSM	1.9 (1.5–2.7)	1.7 (1.2–2.4)*	1.5 (1.2–1.9)	1.5 (1.1–1.9)*
Race/ethnicity				
Caucasian	1.0	1.0	1.0	1.0
African American	0.4 (0.3–0.5)	0.6 (0.5–0.8)**	0.6 (0.5–0.6)	0.7 (0.6–0.9)*
**Clinical**				
ART Adherence				
Adherent	1.0	1.0	1.0	1.0
Non-adherent	0.8 (0.5–1.2)	0.9 (0.6–1.5)	1.5 (1.1–1.9)	1.1 (0.7–1.6)
*Comorbidities* b				
Cardiovascular	1.1 (0.9–1.3)	0.9 (0.8–1.3)	0.7 (0.5–0.8)	0.8 (0.6–0.9)*
Respiratory	1.0 (0.8–1.4)	1.0 (0.7–1.4)	1.3 (1.1–1.6)	1.5 (1.2–1.9)*
Metabolic	1.3 (1.1–1.6)	0.9 (0.7–1.2)	0.7 (0.6–0.9)	0.8 (0.6–0.9)*
Any cancer	1.4 (1.0–1.9)	0.9 (0.6–1.4)	0.8 (0.6–1.1)	0.7 (0.5–1.1)
*Laboratory parameters* c				
Plasma HIV-1 (copies/mL)				
Undetectable (<50)	1.0	1.0	1.0	1.0
Detectable (≥50)	0.9 (0.7–1.2)	1.2 (0.8–1.6)	1.6 (1.3–1.9)	1.5 (1.1–1.9)*
CD4 count (cells/μL)				
<200	1.0	1.0	1.0	1.0
200–350	1.3 (0.8–2.0)	0.9 (0.6–1.4)	0.9 (0.7–1.2)	0.9 (0.6–1.2)
>350	1.2 (0.8–1.7)	0.7 (0.5–1.2)	1.0 (0.7–1.3)	0.8 (0.5–1.2)
**Psychosocial**				
*Depression*				
Not depressed	1.0	1.0	1.0	1.0
Depressed	1.4 (1.1–1.7)	1.2 (0.9–1.5)	2.3 (1.9–2.8)	1.6 (1.3–2.0)**
*Anxiety*				
No anxiety	1.0	1.0	1.0	1.0
Anxiety	1.7 (1.3–2.2)	1.5 (1.1–2.0)*	2.4 (1.9–2.9)	1.6 (1.2–2.1)*
*Substance use*^d^				
Never	1.0	1.0	1.0	1.0
Prior	4.3 (3.3–5.6)	3.2 (2.5–4.3)**	5.6 (4.5–7.1)	4.7 (3.6–6.1)**
Current	3.0 (1.8–5.2)	2.6 (1.4–4.6)*	11.0 (7.2–16.8)	8.3(5.3–13.3)**

Abbreviations: AIDS- Acquired Immunodeficiency Syndrome; ART- Antiretroviral Therapy; CI- Confidence Interval; HIV- Human Immunodeficiency Virus; MSM- Men who have sex with men; UAB- University of Alabama, Birmingham

All the variables were included in the adjusted model. Boldface indicates statistical significance:

* P<0.05 levels,

** P<0.001. Non-smokers were used as the reference group.

All the variables were added in the adjusted model

a Odds ratio per 10-year increment;

b comorbidities: Cardiovascular (stroke, myocardial infarction, cardiovascular disease, hypertension); Respiratory (asthma, chronic obstructive pulmonary disease, bacterial pneumonia); Metabolic (diabetes and dyslipidemia), patients without comorbidities were used as the reference group;

c Lab value closest on or prior to index visit;

d substance abuse (opioids, marijuana, crack/cocaine, amphetamine)
